# Isolated gastric Kaposi sarcoma following allogeneic 2 hematopoietic stem cell transplantation: A Case Report and Literature Review

**DOI:** 10.3389/fmed.2026.1962801

**Published:** 2026-09-18

**Authors:** Aysheh Obeidat, Leen Al-Kraimeen, Fareed Barakat, Zaid Abdel Rahman

**Affiliations:** 1 Department of Internal Medicine, King Hussein Cancer Center, Amman, Jordan; 2 Department of Pathology and Laboratory Medicine, King Hussein Cancer Center, Amman, Jordan; 3 Department of Internal Medicine, King Hussein Cancer Center School of Medicine, University of Jordan, Amman, Jordan

**Keywords:** case report, HHV-8, HSCT, kaposi sarcoma, pneumonitis

## Abstract

**Background:**

Kaposi sarcoma (KS) is a rare complication after hematopoietic stem cell transplantation (HSCT).

**Case presentation:**

We report a 20-year-old male with severe aplastic anemia who underwent HLA-matched sibling allogeneic HSCT. Three months later, he developed abdominal pain, nausea, and diarrhea suspected to represent gastrointestinal graft-versus-host disease. Endoscopic evaluation revealed multiple ulcerated gastric nodules, and histopathologic examination demonstrated spindle-cell proliferation with positive HHV-8 and CD34 immunostaining, confirming KS. Staging with computed tomography of the chest, abdomen, and pelvis showed no additional visceral involvement, and examination of the skin and mucous membranes showed no evidence of cutaneous or mucosal disease. Immunosuppression was gradually tapered. However, the patient developed rapidly progressive respiratory failure with positive blood HHV-8 polymerase chain reaction. HHV-8 pneumonitis was suspected but could not be confirmed, and the patient died despite supportive management.

**Results:**

A literature review identified 20 reported cases of KS following HSCT. Most cases occurred within the first year after transplantation while on immunosuppression. Cutaneous involvement was the predominant presentation, whereas isolated visceral disease was exceptionally uncommon. Reduction or discontinuation of immunosuppression was the most frequent treatment option, while visceral involvement was associated with poorer outcomes.

**Conclusion:**

To our knowledge, no previously published case of isolated gastric KS following allogeneic HSCT was identified in our literature search. It highlights the importance of considering KS in the differential diagnosis of gastrointestinal symptoms after HSCT and supports early endoscopic biopsy with HHV-8 immunohistochemical evaluation to facilitate timely diagnosis.

## Introduction

Kaposi sarcoma (KS) was first described in 1872 by Moritz Kaposi as “idiopathisches multiples Pigmentsarkom,” a rare vascular tumor observed predominantly in elderly men of Mediterranean origin (1). KS is now recognized as an angioproliferative neoplasm characterized by mucocutaneous and visceral lesions and is strongly associated with human herpesvirus 8 (HHV-8), also known as Kaposi sarcoma-associated herpesvirus (KSHV) (2).

Four clinicopathologic forms of KS have been described: classic, endemic (African), epidemic (HIV-associated), and iatrogenic disease (3). Classic KS typically affects older individuals from Mediterranean and Eastern European populations and has been associated with prior HHV-8 infection, increasing age, and environmental factors including exposure to volcanic soil and insect bites (4, 5). Endemic KS demonstrates a more aggressive subtype occurring in HIV-negative individuals in sub-Saharan Africa, with incidence rising dramatically following the HIV epidemic (6). In parallel, HIV-associated KS became one of the most common malignancies in regions heavily affected by HIV infection, particularly in sub-Saharan Africa where HHV-8 seroprevalence is high (7).

Iatrogenic KS occurs in the setting of immunosuppression, most commonly after solid organ transplantation. Many factors have been associated with an increased risk of post-transplant KS, including male sex, nonwhite ethnicity, older age, HLA mismatch, and the duration of immunosuppressive therapy (8). In contrast, Kaposi sarcoma following hematopoietic stem cell transplantation (HSCT) remains exceedingly rare, with only a limited number of cases reported in the literature.

Herein, we report a unique case of isolated gastric Kaposi sarcoma developing after allogeneic HSCT in the absence of cutaneous, mucosal, or nodal disease. We additionally review the published literature on post-HSCT Kaposi sarcoma, highlighting clinical presentation, treatment approaches, and outcomes. To our knowledge, no previously published case of isolated gastric KS following allogeneic HSCT was identified in our literature search.

## Case description

### Patient information

A 20-year-old male with no significant past medical history was found to have severe pancytopenia during routine laboratory evaluation and was subsequently diagnosed with severe aplastic anemia. The patient was referred to our center for full work-up and preparation for allogeneic hematopoietic stem cell transplantation (HSCT). Extensive evaluation did not identify an underlying cause; the patient had negative serologic testing for viral hepatitis B, C, human immunodeficiency virus (HIV), cytomegalovirus (CMV), and Epstein–Barr virus (EBV). Pre-transplant HHV-8 serology/PCR was not performed in either the recipient or donor. Chromosomal breakage study was negative, and high-sensitivity flow cytometry to detect the absence of GPI-anchored proteins (CD55/CD59) was also negative. Bone marrow biopsy confirmed the diagnosis, showing a cellularity of 2% with marked reduction in granulocytic, erythroid, and megakaryocytic precursors, consistent with a diagnosis of severe aplastic anemia. A 54-gene myeloid panel did not identify any somatic mutation.

### Initial therapeutic intervention

His pre-transplant course was complicated by severe cellulitis of his left upper extremity, which was managed with broad-spectrum intravenous antibiotics and local care. He subsequently received an allogeneic HSCT from a matched sibling donor using a myeloablative regimen of cyclophosphamide (50 mg/kg adjusted ideal body weight intravenously on days −5, −4, −3 and −2) and rabbit anti-thymocyte globulin (r-ATG) at a dose of 2.5 mg/kg actual body weight on days −3, −2 and −1. On day 0, the patient received a fresh infusion of 6.63 × 10⁶ CD34 + cells/kg recipient body weight. Tacrolimus was initiated on day −2 for graft-versus-host disease (GVHD) prophylaxis.

His post-transplant course was complicated by a perianal fistula, which was managed conservatively with antimicrobial therapy and local wound care, and two episodes of CMV reactivation managed preemptively with valganciclovir. Neutrophil and platelet engraftment occurred by day +15, with subsequent transfusion and growth factor independence. Chimerism testing on day +30 showed full donor chimerism.

### Clinical presentation

Three months after transplantation, the patient developed mild upper abdominal pain, nausea, and diarrhea. Given the concern for gastrointestinal GVHD, upper and lower endoscopic evaluation was performed. Empiric prednisone (2 mg/kg/day) was initiated.

### Diagnostic assessment

Sigmoidoscopy showed grossly normal rectosigmoid mucosa; histopathological examination revealed mild graft-versus-host disease with a negative CMV immunohistochemical stain. Esophagogastroduodenoscopy (EGD) showed nodular gastric mucosa with six red nodules with an ulcerated surface, the largest measuring 8 × 8 mm. A biopsy of these nodules revealed no histologic evidence of GVHD. Histopathologic examination demonstrated spindle-cell proliferation arranged in fascicles with slit-like vascular spaces and extravasated erythrocytes (Figure 1A). Immunohistochemical staining showed positivity for CD34 and HHV-8 latent nuclear antigen-1, and negativity for DOG1, S100, and Desmin, confirming the diagnosis of Kaposi sarcoma (Figures 1B,C). At the time of KS diagnosis, staging with CT of the chest, abdomen, and pelvis showed no evidence of additional visceral involvement. Examination of the skin and mucous membranes showed no evidence of cutaneous or mucosal KS.

**Figure 1:**
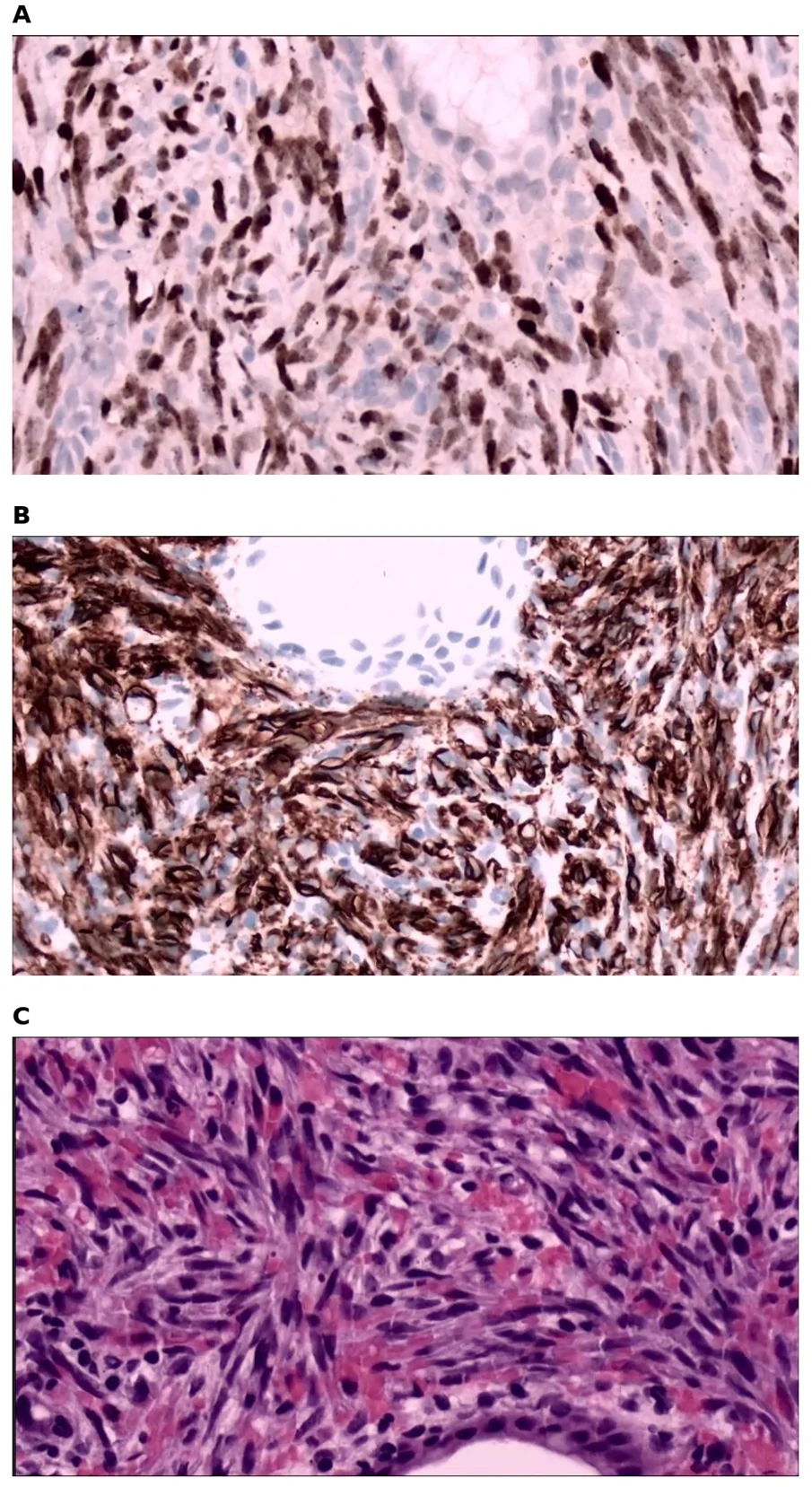
Histopathologic annd immunohistochemical features of gastric kaposi sarcoma. **(A)** HHV-8 latency-associated nuclear antigen-1 (LNA-1) immunostaining showing diffuse nuclear positivity in the spindle cells. **(B)** CD34 immunostaining showing diffuse positivity in the spindle cells (original magnification  × 40). **(C)** Hematoxylin and eosin (H&E)-stained section showing a fascicular spindle-cell proliferation with slit-like vascular spaces and extravasated erythrocytes (original magnification  × 40).

### Therapeutic intervention

Given the limited disease burden and isolated gastric involvement at the time of diagnosis, reduction of immunosuppression was selected as the initial management strategy.

### Follow-up and outcomes

Two weeks after his diagnosis, the patient was admitted with progressive respiratory distress, fever, and cough. Chest radiography demonstrated progressive bilateral pulmonary consolidation. Extensive infectious work-up was unrevealing except for a positive HHV-8 PCR in peripheral blood, and he started on foscarnet intravenously. His respiratory status deteriorated rapidly over the following 48 h, progressing to acute respiratory distress syndrome requiring mechanical ventilation. HHV-8 pneumonitis was suspected in the setting of progressive pulmonary disease and detectable HHV-8 DNA in peripheral blood; however, this diagnosis could not be confirmed in the absence of pulmonary tissue or other definitive diagnostic evidence. Despite supportive management, the patient died a few days later. The patient's clinical course is summarized in Table 1.

**Table 1:** Timeline of the patient's clinical course.

Time	Event
Day 0	Allogeneic HSCT
Day +15	Engraftment
Day +30	Full donor chimerism
After 3 months	Developed abdominal pain, nausea, diarrhea
After 3 months	Underwent endoscopy
After 3 months	Diagnosed with gastric KS
After 3 months	Immunosupression tapered
2 weeks post KS diagnosis	Developed respiratory failure with suspected HHV-8 pneumonitis
Few days later	Death

## Literature search methods

A literature search was performed to identify previously reported cases of Kaposi sarcoma following hematopoietic stem cell transplantation. PubMed/MEDLINE and Embase were searched using combinations of the terms “Kaposi sarcoma,” “Kaposi's sarcoma,’ “HHV-8,” “human herpesvirus 8,” “hematopoietic stem cell transplantation,” “hematopoietic stem cell transplant,” “bone marrow transplantation,” and “allogeneic stem cell transplantation.” The search was conducted in March 2026. Published case reports and case series describing KS following HSCT were eligible for inclusion. Reports of KS unrelated to HSCT, studies involving solid-organ transplantation only, non-human studies, and publications without sufficient clinical information to characterize the KS presentation were excluded. Reference lists of relevant articles were also reviewed to identify additional cases.

## Literature review and discussion

Kaposi sarcoma (KS) is traditionally classified into four clinicopathologic subtypes: classic, endemic, epidemic (HIV-associated), and iatrogenic disease (9). Iatrogenic KS most commonly occurs in the setting of prolonged immunosuppression, particularly after solid organ transplantation, with the highest incidence reported among kidney transplant recipients (10). In our case and literature review, we describe iatrogenic Kaposi sarcoma occurring after HSCT.

Among the 20 published cases identified in our review, most occurred within the first year following HSCT. However, these findings should be interpreted cautiously because the available evidence is limited to a small number of published cases and is subject to publication bias. The time from transplant to KS development ranged from 1.5 to 39 months, with a median of 7 months. Approximately 40% of patients developed the disease within 6 months after HSCT. Most cases occurred during periods of active immunosuppression, supporting the role of impaired immune surveillance in HHV-8 reactivation. There was a strong male predominance in reported cases, with a male-to-female ratio of 2.3:1. This was even more pronounced among adults.

In our literature review, a total of 20 patients were identified: eight pediatric cases (11–17) and 12 adult cases (18–29) as summarized in Table 2 and Table 3. Patients' mean age was approximately 9 years in pediatric cases (range 5–17 years) and 47 years in adults (range 26–61 years). In both groups, the most common presentation was cutaneous lesions, appearing as violaceous skin nodules or plaques (15 out of 20 cases, 75%). Lymph node involvement was documented in 30% of patients. Mucosal involvement (oral cavity, gingiva, palate) was reported in approximately 40% of the patients. Visceral involvement was less common but occurred more frequently in pediatric patients. Pediatric cases appeared more likely to demonstrate visceral dissemination, particularly in the lung and gastrointestinal tract (75% in pediatric cases compared to 33% of adult cases).

**Table 2:** Published pediatric cases of kaposi sarcoma following hematopoietic stem cell transplantation.

Author, year	Age/sex	Underlying disease	HSCT type	Time to KS	Site of KS	Immunosuppression	HHV-8 testing	Treatment	Outcome
Abbas et al., 2012	8/M	Relapsed AML	Matched related allo-HSCT	8 months	Gingiva, skin, respiratory tract	Cyclosporine, prednisone	Positive IHC	IS withdrawal	Alive
Porta et al., 1991	7/M	Lymphoblastic NHL	Autologous HSCT	5 months	Lymph nodes	None	NR	Interferon-α	Alive
Wang et al., 2023	6/F	Severe aplastic anemia	Haploidentical allo-HSCT	11 months	Skin, oral mucosa, GI tract	Cyclosporine, prednisone	Positive IHC	Cyclosporine taper	Alive
Alsaidan et al., 2023	11/M	Fanconi anemia	Allogeneic HSCT	6.5 months	Skin, oral cavity, liver	Sirolimus, tacrolimus, corticosteroids	Positive IHC	IS taper	Alive
Sidorov et al., 2023	5/F	ALL	Haploidentical allo-HSCT	NR	Isolated lung	NR	Positive IHC	Liposomal doxorubicin	NR
Sidorov et al., 2023	17/M	Kabuki syndrome	Matched unrelated allo-HSCT	Few months	Lymph node	None	Positive IHC	Liposomal doxorubicin	Deceased
Valero-Arrese et al., 2021	10/M	Severe aplastic anemia	Matched sibling allo-HSCT	4.8 months	Skin, mucosa, lymph nodes, lungs	MMF	Positive IHC	MMF withdrawal	Alive
Sala et al., 2011	10/M	AML	NR	15.4 months	Mucosa, tonsils, lymph nodes, lungs, pancreas, biliary tree, sinuses	Cyclosporine, AZA, methylprednisolone	Positive IHC	IS withdrawal + liposomal doxorubicin	Alive

ALL, acute lymphoblastic leukemia; AML, acute myeloid leukemia; allo-HSCT, allogeneic hematopoietic stem cell transplantation; GI, gastrointestinal; HHV-8, human herpesvirus 8; HSCT, hematopoietic stem cell transplantation; IHC, immunohistochemistry; IS, immunosuppression; KS, Kaposi sarcoma; MMF, mycophenolate mofetil; NHL, non-Hodgkin lymphoma; NR, not reported.

**Table 3:** Published adult cases of kaposi sarcoma following hematopoietic stem cell transplantation.

Author, year	Age/sex	Underlying disease	HSCT type	Time to KS	Site of KS	Immunosuppression	HHV-8 testing	Treatment	Outcome
Zhou et al., 2022	29/M	AML	Haploidentical allo-HSCT	7.6 months	Skin, oral cavity, lungs, GI tract	Cyclosporine, prednisone	Positive IHC	Sirolimus + liposomal doxorubicin	Alive
Innes et al., 2017	58/M	Peripheral T-cell lymphoma	Reduced-intensity allo-HSCT	22 months	Skin only	Multiple agents	Positive IHC	Chemotherapy	Deceased
Geller et al., 2018	52/M	Mantle cell lymphoma	Autologous HSCT	5 months	Skin, hard palate	Rituximab	Positive IHC	Rituximab discontinued	Alive
Ye et al., 2011	33/M	Severe aplastic anemia	Matched sibling allo-HSCT	6.6 months	Lymph nodes, skin, peri-orbital tissue	Prednisone	Positive IHC	No treatment	Deceased
Chen et al., 2025	61/M	VEXAS syndrome	Matched unrelated allo-HSCT	39.1 months	Oral cavity, esophagus	Cyclosporine, prednisone	Positive IHC	Liposomal doxorubicin	Alive
Heyrman et al., 2016	61/M	Primary mediastinal NHL	Tandem autologous HSCT	1.5 months	Skin only	None	Positive IHC	Observation	Alive
Ramzi et al., 2021	44/F	AML	Haploidentical allo-HSCT	4 months	Upper lip lesion	Cyclosporine, prednisone	Positive IHC	Cyclosporine withdrawal + cryotherapy	Alive
de Medeiros et al., 2000	46/F	CML	Matched sibling allo-HSCT	27.1 months	Skin only	Azathioprine, prednisone	Positive IHC	IS taper + radiotherapy + thalidomide	Alive
Erer et al., 1997	26/M	Sickle-thalassemia	Matched sibling allo-HSCT	29 months	Skin only	Cyclosporine, methylprednisolone	NR	IS withdrawal	Alive
Avivi et al., 2011	46/M	CML	T-cell depleted allo-HSCT	6 months	Skin, mucosa, lung, liver, oropharynx	Prednisone	NR	Interferon + low-dose doxorubicin	Deceased
Gluckman et al., 1995	Female	AML	Matched sibling allo-HSCT	8 months	Skin, mucosa, esophagus, stomach	NR	Positive blood PCR + tissue DNA	Interferon + radiotherapy	Deceased
Laspa et al., 2025	61/F	AML	Haploidentical allo-HSCT	4.1 months	Skin, lymph nodes	Tacrolimus	Positive blood PCR + IHC	Liposomal doxorubicin + rituximab	Alive

AML, acute myeloid leukemia; allo-HSCT, allogeneic hematopoietic stem cell transplantation; CML, chronic myeloid leukemia; GI, gastrointestinal; HHV-8, human herpesvirus 8; HSCT, hematopoietic stem cell transplantation; IHC, immunohistochemistry; IS, immunosuppression; KS, Kaposi sarcoma; NHL, non-Hodgkin lymphoma; NR, not reported; VEXAS, vacuoles, E1 enzyme, X-linked, autoinflammatory, somatic syndrome.

Human herpesvirus 8 reactivation is a well-established driver in the pathogenesis of Kaposi sarcoma, as well as other tumors such as primary effusion lymphoma and multicentric Castleman disease (MCD) (30). While HHV-8-associated tumors are most common in patients with HIV (31), they have also been reported in other immunosuppressed individuals. Among the 20 cases reviewed, HHV-8 was detected in 16 patients using immunohistochemical (IHC) staining on tissue biopsy. HHV-8 PCR in the blood was performed in only 6 patients and was detected in 3 cases (50%). Interestingly, blood HHV-8 PCR positivity appeared more common among patients with visceral disease.

The source of HHV-8 infection in post-HSCT KS remains uncertain and may reflect either recipient viral reactivation or donor-derived transmission. Routine HHV-8 screening is not universally performed before HSCT, particularly in regions with lower endemicity.

The development of KS in transplant recipients is believed to result from impaired T-cell–mediated immune surveillance, allowing latent HHV-8 reactivation and subsequent endothelial proliferation and angiogenic signaling (32). In our literature review, calcineurin inhibitors (CNIs), particularly cyclosporine A, were the most commonly used immunosuppressants at the time of KS diagnosis, used in 10 of 20 reported cases (50%). Additionally, approximately half of patients were receiving corticosteroids at the onset of KS. Antimetabolites such as mycophenolate and azathioprine were in use in only 2 cases. Notably, sirolimus was not implicated as a causative agent in any of the reported cases; in fact, it is a recognized antiangiogenic agent (33) and was used for treatment in two cases after KS diagnosis (one patient was already on sirolimus and continued it during treatment, and one patient on cyclosporine A was switched to sirolimus when KS was diagnosed). In both cases, marked regression of disease was noted. Only three of our reviewed cases developed KS after autologous HSCT (15% of cases). The remaining cases occurred after allogeneic HSCT, likely reflecting the greater intensity and duration of immunosuppression required for GVHD prophylaxis and treatment.

Given the absence of treatments capable of eradicating latent HHV-8 infection, the first-line treatment for iatrogenic KS is immunosuppression withdrawal. Chemotherapy, particularly liposomal doxorubicin, is usually reserved for disseminated or refractory disease. Local therapies, interferon-alpha, and radiotherapy have also been utilized in selected cases (33). In our literature review, 13 of 20 reported cases had immunosuppression tapering or discontinuation, resulting in a clinical response (partial or complete regression) in around 60% of cases. Most patients who responded showed improvement within the first 2 months. Systemic chemotherapy was administered in 8 cases (40%), with liposomal doxorubicin being the most commonly used agent; two of the eight cases achieved complete resolution. Interferon-alpha was used in two reported adult cases and one pediatric case, possibly due to the inability to administer chemotherapy early in the post-HSCT course. Among those treated with interferon-alpha, one case with only cutaneous involvement showed a complete response within eight months, while the other two with disseminated/visceral KS, showed disease progression.

The prognosis for iatrogenic Kaposi sarcoma is generally considered poorer than other types of KS (9). This is likely related to the degree of underlying immune dysfunction and the challenges associated with reducing immunosuppressive therapy. Outcome in post-transplant KS depends on the site of disease involvement, comorbidities, and the extent of immunosuppression used. In our literature review, KS with only cutaneous involvement had the most favorable prognosis, with approximately 89% of reported cases achieving clinical response after immunosuppression withdrawal. Reported cases with combined cutaneous and mucosal involvement showed mixed responses, with a significant number requiring additional therapy beyond immunosuppression withdrawal. In contrast, visceral KS carried the worst prognosis, with over one third of the reviewed cases reporting death as the outcome. Nearly 60% of visceral KS cases received systemic chemotherapy.

Our case is unique because, to our knowledge, no previously published case of isolated gastric KS following allogeneic HSCT without cutaneous, mucosal, or lymph node involvement was identified in our literature search. Consistent with the literature review, our patient developed KS while receiving immunosuppression with calcineurin inhibitors, with the recent addition of steroids for management of GVHD. Notably, the ATG-based myeloablative conditioning used for transplant may have further contributed to the pathogenesis of KS. While no published study quantifies the risk of KS by conditioning regimen *per se*, published reports suggest that myeloablative conditioning results in a state of profound T-cell suppression that may contribute to latent HHV-8 reactivation (26, 34). In our patient, the onset of KS was nearly 4 months post-HSCT, similar to nearly 40% of the reviewed cases. No cutaneous lesions or oral mucosal involvement were identified on repeated physical examination. The subsequent development of rapidly progressive respiratory failure raises the possibility of pulmonary KS or progression to disseminated disease. However, pulmonary KS could not be confirmed. Therefore, the designation of isolated gastric KS in this report refers specifically to the extent of disease demonstrated at the time of initial diagnosis and staging. Despite tapering immunosuppression and supportive management, the patient developed rapidly progressive respiratory failure. HHV-8 pneumonitis was suspected but could not be definitively confirmed.

## Conclusion

Kaposi sarcoma is an uncommon but important complication following HSCT, typically driven by immune dysfunction and HHV-8 reactivation. Most reported cases present with cutaneous or mucosal disease, often within the first year after transplant, and frequently occur in patients receiving calcineurin inhibitors. Isolated visceral involvement is extremely rare. To our knowledge, no previously published case of isolated gastric Kaposi sarcoma following allogeneic HSCT was identified in our literature search. This underscores the need for a high index of suspicion when evaluating gastrointestinal symptoms in HSCT recipients, even in the absence of skin lesions. Early endoscopic evaluation with histopathologic examination and HHV-8 immunohistochemical staining remains essential for diagnosis. While immunosuppression reduction is the cornerstone of management, outcomes are largely influenced by the degree of underlying immune dysfunction. In our patient, rapidly progressive respiratory failure developed shortly after the diagnosis of gastric KS; the underlying pulmonary process remained unconfirmed. Greater awareness of this rare presentation may facilitate earlier detection and improved outcomes.

## Limitations

This report is limited by its single-patient design, the lack of definitive characterization of the subsequent pulmonary process, and the limited availability of pre-transplant HHV-8 virologic data. In particular, pulmonary KS or progression to disseminated disease could not be definitively excluded. Nevertheless, the detailed clinicopathologic characterization and comprehensive literature review provide valuable insights into this exceptionally rare complication.

## Patient perspective

A patient perspective could not be obtained because the patient died shortly after the events described. Despite reasonable efforts, the patient's next of kin could not be contacted.

## Data Availability

The original contributions presented in the study are included in the article/Supplementary Material, further inquiries can be directed to the corresponding author.
